# Supplementary material for: Investigating the experiences of low-carbohydrate diets for people living with Type 2 Diabetes: A thematic analysis
Source: PLoS One. 2022 Aug 22;17(8):e0273422. doi: 10.1371/journal.pone.0273422 (PMC9394793; doi:10.1371/journal.pone.0273422)
Supplement: Attachment — Submitted filename: Response to Reviewers.docx [file pone.0273422.s002.docx]

**Response to Reviewers**	5^th^ July 2022

Dear Editor and reviewers

Thank you for your helpful and constructive comments, greatly appreciated. We have responded to these as follows:

***Editor#:*** *The current manuscript outlined the experiences of low-carbohydrate diets for people living with Type 2 Diabetes. The research interest if good. However, some important bottlenecks are pending to be improved to further clarify the results justifications. Kindly take note that a human ethical approval from institutional review board is mandatory (authors are required to provide the approval number). Also, the manuscript is recommended to add elements such as strengths and limitations.*

Thank you, we have responded to the reviewer specific points below. Specifically we have responded to the comments on the ‘quantitative’ based queries and have added these in to the limitations of the study, and we have amended some language in the findings to clarify these are self-reported clinical improvements in line with qualitative methodology. We have clarified the ethical approval within the manuscript and added strengths into the now strengths and limitations section of the discussion. We have reviewed the quality of figure 1 (and checked this using PACE tool) and we have checked the format of the manuscript against the journal guidelines. We have un-blinded the manuscript and added author initials etc. where appropriate.

***Reviewer #1:*** *Type 2 diabetes negatively affects the mental health as well as the physical health of the individual, especially in the long term. We need to learn how patients feel about their illness and care about it. The number of such studies should definitely be increased. I congratulate you for your great work !*

Thank you for your comments.

***Reviewer #2****: 1. As it is qualitative research kindly justify how can you ensure participants gave proper answer your online interview*

Thank you for your comment. We believe we have provided a detailed description of our data collection process in line with qualitative, thematic analysis methodology. As describe the researcher built rapport with the participants and then the interviews were recorded, and later transcribed. Participants were given every opportunity discuss and answer questions, as described within the methodology:

“Semi-structured interviews allowed for detailed and personal discussions surrounding the participants' experience of LCDs. The use of open-ended questions such as "How have you found maintaining your LCD?" gave the participants the opportunity to discuss aspects important to them. This also allowed the researcher to follow up any themes of interest that developed from the discussions. An interview schedule was used as a basis to guide the semi-structured interviews”.

“ Prompt questions were also included to ensure that the interview imitated a 'flowing conversation' [44].”

***Reviewer #2.***

***2.*** *As you mentioned in the theme-I lack of professional guidance for dietary management of T2DM, How can you ensure they following LCD diets and have you calculated their carbohydrates intake or study participants knows carbohydrates counting.*
***3****. As you mentioned in the theme-IV LCD had improved their T2DM and HbA1c dropped significantly. have you analyzed their pre post HbA1c level and other blood profiles*

We have clarified the language in the theme 4 to be more specific that these changes were self-reported.

For example “All respondents *reported* that their HbA1c levels had dropped significantly”

Regards points 2 & 3- we have added the following into the strengths and limitations section of the discussion to acknowledge these points:

“As a qualitative study, the methodology did not seek to quantify the participants LCD or to assess changes in diabetes clinical outcomes (e.g. HbA1c), rather it sought to explore their perceptions and experiences of choosing a LCD. Further research to explore the implementation of LCD in practice, and to assess changes in clinical outcomes for diabetes is warranted.”

***Reviewer #3:*** *Newson and Parody sought to explore the experiences of participants living with Type 2 Diabetes, utilizing a Low-Carbohydrate diet. Participants reported gaining knowledge and skills to increase their Capability to engage in LCDs. Motivation to manage diabetes outcomes influenced adherence. However, challenges were reported with the Opportunity to engage in behavior, mainly influenced by social support. The manuscript was generally technically sound, and the data support the conclusions. The thematic analysis was very well outlined and very coherent. The written English was clear and very standard.*

Thank you for these comments

*Strengths of the study. The study area is an emerging one with rising interest. This study serves as a useful baseline study that sought to utilize a psychological investigation to understand lay people's perceptions of LCDs and their reasoning and motivations for engaging in such a diet. Also, the research also focused on how people could sustain their LCD, how they managed it, and also explored potential barriers or challenges they faced. In addition, the study also explored the perceived effects of LCDs on people's T2DM and their general health and well-being.*

We have added a strengths sections specifically into the discussion, and utilised your feedback to assist with this.

*Limitations of the study were well outlined by the authors.*

Thank you
 *General comments
1. Ethics approval number and the institution that granted the ethics were not indicated--- line 180*

Ethical approval details have now been confirmed within the manuscript itself.

*2. Figure 1 quality appears to be low. Authors should submit high resolution figure if manuscript is accepted eventually.*

We have reviewed the Figure 1 quality again, thank you. We have checked this with PACE tool for quality, thank you.

*Other corrections and suggestions have been uploaded-*

Thank you, we have made the four minor amends to the transcript as suggested via the attached upload.

We hope this satisfies the review and welcome your response.

Many thanks

Dr Newson
